# The Imperative of Palliation in the Management of Rabies Encephalomyelitis

**DOI:** 10.3390/tropicalmed2040052

**Published:** 2017-10-04

**Authors:** Mary J. Warrell, David A. Warrell, Arnaud Tarantola

**Affiliations:** 1 Oxford Vaccine Group, University of Oxford, Centre for Clinical Vaccinology & Tropical Medicine, Churchill Hospital, Old Rd, Headington, Oxford, OX3 7LJ, UK; 2 Nuffield Department of Clinical Medicine, University of Oxford, John Radcliffe Hospital, Oxford, OX3 9DW, UK; david.warrell@ndm.ox.ac.uk; 3 Institut Pasteur de Nouvelle-Calédonie, BP 61 – 98845 Nouméa cedex, New Caledonia; atarantola@pasteur.nc

**Keywords:** rabies, encephalomyelitis, palliation, diagnosis, treatment

## Abstract

The aim of this review is to guide clinicians in the practical management of patients suffering from rabies encephalomyelitis. This condition is eminently preventable by modern post-exposure vaccination, but is virtually always fatal in unvaccinated people. In the absence of any proven effective antiviral or other treatment, palliative care is an imperative to minimise suffering. Suspicion of rabies encephalomyelitis depends on recognising the classic symptomatology and eliciting a history of exposure to a possibly rabid mammal. Potentially treatable differential diagnoses must be eliminated, notably other infective encephalopathies. Laboratory confirmation of suspected rabies is not usually possible in many endemic areas, but is essential for public health surveillance. In a disease as agonising and terrifying as rabies encephalomyelitis, alleviation of distressing symptoms is the primary concern and overriding responsibility of medical staff. Calm, quiet conditions should be created, allowing relatives to communicate with the dying patient in safety and privacy. Palliative management must address thirst and dehydration, fever, anxiety, fear, restlessness, agitation, seizures, hypersecretion, and pain. As the infection progresses, coma and respiratory, cardiovascular, neurological, endocrine, or gastrointestinal complications will eventually ensue. When the facilities exist, the possibility of intensive care may arise, but although some patients may survive, they will be left with severe neurological sequelae. Recovery from rabies is extremely rare, and heroic measures with intensive care should be considered only in patients who have been previously vaccinated, develop rabies antibody within the first week of illness, or were infected by an American bat rabies virus. However, in most cases, clinicians must have the courage to offer compassionate palliation whenever the diagnosis of rabies encephalomyelitis is inescapable.

## 1. Introduction

The primary aim of this review is to re-emphasise the humanitarian role of palliative treatment in the compassionate care of patients with rabies encephalomyelitis, who have no chance of recovery. We provide practical details of this management and summarise the clinical and laboratory evidence leading to the fateful diagnosis of rabies encephalomyelitis.

Rabies infection following bites by dogs and other terrestrial mammals is eminently preventable by full modern post-exposure treatment, but if this opportunity has been missed and the virus has infected the nervous system, the condition is almost always fatal in unvaccinated people. Two children bitten by bats in the USA, only one of whom had received rabies post-exposure vaccination, have recovered from rabies encephalomyelitis to live independent lives [1,2]. Another person, who was bitten by a dog in Turkey and was incompletely vaccinated, also recovered [3]. However, most rabies patients given expert intensive care have either died in the acute phase or have survived for varying periods of time, but with incapacitating neurological impairment. [4,5]. In the absence of any proven effective antiviral or other treatment, how should clinicians manage patients with this appalling disease, particularly in poorer countries, where dog rabies is endemic?

The practical management of patients in this dire situation has been neglected, while thousands of patients continue to die each year after terrible suffering. For example, in India, 12,700 symptomatically identifiable furious rabies deaths occur annually, 41% in children under the age of 15 years [6]. However, patients dying of paralytic or dumb rabies are much more difficult to diagnose clinically and therefore to enumerate. No practical advice about the clinical management of rabies patients is available in dog rabies-endemic areas, where palliative care is the best and only option. As a result, clinicians presented with the occasional rabies patient have been forced to improvise. They feel unable to cope with the unpredictable, sometimes agitated, behaviour typical of ‘furious’ rabies encephalomyelitis. As a result, patients have been abandoned in isolated rooms and bound to their beds. The hydrophobic spasms of furious rabies are associated with feelings of terror and great agitation, and some patients may become aggressive [7]. Relatives needing an opportunity to ask questions and comfort the victim are also neglected, and patients may be sent home to families who are unable to deal with their distressing symptoms and are frightened of being infected themselves.

## 2. Clinical Recognition of Rabies Encephalomyelitis

The incubation period may be highly variable, but is usually 20–60 days after the bite exposure. Itching or other paraesthesiae at the site of the healed bite wound are common prodromal symptoms. However, many patients present with non-specific fever, headache, myalgia, fatigue, sore throat, gastrointestinal symptoms, irritability, anxiety, insomnia, or even hallucinations and may be referred to a wide variety of specialists. Correct diagnosis depends on taking a full history that may reveal the possibility of exposure to a potentially rabid mammal.

Usually within a week, the disease progresses to either furious or paralytic rabies encephalomyelitis [8]. Furious rabies is the commoner and more easily recognisable form. Hydrophobic spasms are pathognomonic and may be the only physical sign in the early stages. Growing thirst forces patients to ask for water, but attempts to drink, the sound of a tap running, or the mere mention of water, a draught of air (aerophobia), touching the palate, bright lights or loud noises may provoke powerful, jerky contractions of the diaphragm and accessory muscles of inspiration, sometimes with gripping retrosternal pain suggesting oesophageal spasms. There is no evidence of the laryngeal spasms and upper airway obstruction implied in some accounts [9]. At the end of the attack, the patient may convulse and become contorted into a position of transient opisthotonos. Patients describe a feeling of terror that they are unable to explain, associated with the spasms. Many die of respiratory or cardiac arrest during these episodes [10]. Phases of extreme excitement, aggression, anxiety, or hallucinations are interspersed with lucid intervals, during which patients may fully comprehend their appalling predicament. Other features include fever, meningism, cranial nerve lesions, autonomic nervous system overactivity (labile temperature, blood pressure, pulse rate, sweating, hypersalivation, lacrimation), cardiac tachyarrhythmias, myocarditis, dramatic polyuria due to diabetes insipidus, haematemesis from Mallory-Weiss oesophageal tears, and hypersexuality. Even with intensive care, patients soon become comatose and may die within a few days or survive for many weeks or months.

The more insidious paralytic or ‘dumb’ form of the disease is far less distinctive and consequently less often recognised [11,12,13]. It is characteristic of vampire bat-transmitted rabies and some cases of failed postexposure vaccination. Prodromal symptoms are followed by paraesthesiae, fever, and flaccid paralysis, starting in the bitten limb and then ascending with fasciculations [14], sensory symptoms, sphincter dysfunction, and terminal bulbar and respiratory paralysis. Hydrophobic spasms and excitation are usually absent. Even without supportive care, these patients may survive as long as 30 days [11].

If rabies is suspected, every effort should be made to eliminate potentially-treatable differential diagnoses, including encephalopathies caused by other viruses, bacteria including rickettsia, fungi, parasites, drugs, toxins, and psychotic states [15]. This point is exemplified by the case of an agitated teenage girl admitted to hospital with a diagnosis of acute psychosis. She then became drowsy and developed seizures and phobic spasms. Rabies encephalitis was diagnosed, but when she was referred to another hospital in coma, investigations revealed *P. falciparum*. After treatment for cerebral malaria, she recovered fully [16]. Conversely, however, among 133 children dying of central nervous system infections in Malawi, 10.5% had unsuspected rabies encephalitis confirmed at post-mortem. Three (11.5%) of 26 of the fatal cases originally diagnosed as cerebral malaria were in fact due to rabies [17].

## 3. Confirmation of Suspected Rabies

This is important to stop the search for a potentially-treatable condition, to decide on a clinical management plan, to warn others possibly infected by the same animal but unvaccinated, and to offer pre-exposure prophylaxis to relatives and caregivers. Positive diagnoses also contribute to public health surveillance, and guide prevention. However, in most countries with enzootic dog rabies, the diagnosis of human rabies is rarely feasible. Animal testing laboratories are scarce and usually found only in major cities. With advances in techniques, the diagnosis could be made at a remote laboratory, or possibly on a post-mortem needle necropsy by a rapid test on-site [18,19,20,21]. Clinicians must be made aware of the possibilities and provide adequate specimens for testing.

### 3.1. Intravitam Confirmation of Human Rabies Encephalitis (Table 1)

The diagnosis of rabies can be made by isolation of virus, identification of antigen or, in unvaccinated patients, antibody detection. First, it is important to find out from the local rabies reference laboratory which tests they can perform, which samples to take, whether to store them in a fridge or frozen, and how to package and send them. Isolation of the virus in tissue culture or by inoculation of suckling mice confirms the diagnosis with high specificity but lower sensitivity than other methods. It is also less technically demanding, uses cheaper reagents, and is the method of choice.

Positive results are most likely during the first week of illness, from saliva, throat, trachea or eye swabs, brain biopsy samples, and CSF. Viraemia has not been detected.

Rabies diagnosis is most commonly made by a variety of reverse transcription polymerase chain reaction (RT-PCR) tests on saliva, skin biopsy or CSF, but this is possible only in specialist laboratories [22]. Remote testing can be very successful. A reverse-transcription hemi-nested polymerase chain reaction (RT-hnPCR) gave excellent results in Cambodia, Madagascar, Senegal, and France when performed on a skin biopsy specimen taken any time after the onset of symptoms, or from at least three successive saliva samples from each patient [23].

A direct immunofluorescent antibody (IFA) test rapidly confirms antigen in frozen sections of skin biopsies taken from a hairy area, usually the nape of the neck [19,24,25,26]. False positives have not been reported. Unfortunately, this test is rarely performed. The corneal smear IFA test is very insensitive, and the results are unreliable [19,26].

In unvaccinated patients, detection of rabies neutralising antibody is diagnostic. This might appear in the second week of illness, but patients often die before generating any antibody response. In vaccinated people, very high and rising levels of antibody in the serum, and especially in the CSF, suggest the diagnosis [1,2].

The two validated serological methods, the fluorescent antibody neutralization test (FAVN) and the rapid fluorescent focus inhibition test (RFFIT), are technically demanding. Commercial ELISA kits are simpler but do not always correlate with the standard tests. Direct IF antibody methods are unreliable at low levels as cross-reactions occur with other viruses [27].

### 3.2. Postmortem Diagnosis

All the methods mentioned above may be used to confirm the diagnosis postmortem, especially if the clinical illness was very short. Brain samples offer the best chance of successful postmortem diagnosis. These can be obtained without a full necropsy examination (Table 1). Other encephalitides or cerebral malaria may also be diagnosed from brain tissue.

In any part of the world, but especially where there are cultural or religious objections to delayed burial or a full autopsy that includes craniotomy, post-mortem needle necropsy is a most useful technique for obtaining brain samples from anterior or posterior fossae and brain stem [28] (Table 1). The usual diagnostic technique is the direct IFA test as used on animals [29]. A local veterinary laboratory may be helpful if the test is done routinely. If no IF microscope is available, the alternative is a direct rapid immunohistochemical test (dRIT) using biotinylated monoclonal antibodies and light microscopy.

Rabies rapid antigen immunodiagnostic test (RIDT) lateral flow kits are being developed for animal testing in the field [20] and could also be used with human brain samples.

## 4. Care of Patients with Confirmed Rabies Encephalomyelitis

In a disease as agonising and terrifying as rabies encephalomyelitis, alleviation of distressing symptoms is the primary concern and overriding responsibility of medical staff. However, in many clinics and hospitals across rabies endemic areas of the world, patients suspected of having rabies are deemed to be untreatable. They are either sent home with their relatives without advice or drugs or are isolated and sometimes abandoned in a remote part of the health facility and denied any medical attention. These practices ignore the fundamental precept that a doctor's responsibility is to relieve suffering even if there is no expectation of cure.

### 4.1. Accommodation

To avoid provoking hydrophobic or aerophobic spasms, calm, quiet conditions should be created, ideally in a dimly-lit, draught-free, single-bedded room. Restraining the patient in bed can be attempted initially with loose and comfortable ties and cot-sides, and ultimately by adequate sedation. Relatives must be able to communicate with the dying patient with dignity, in safety and privacy, according to their cultural and religious traditions. Other visitors should be restricted, including hospital staff not directly involved in management. However, frequent monitoring is needed so that patients can be given adequate supportive treatment.

### 4.2. Protection of Staff, Relatives, and Other Patients

Although there is no virologically documented instance of person-to-person rabies transmission, patients’ saliva and secretions may contain the virus, and so they should be isolated, and their relatives and medical attendants protected with gloves, masks, and gowns, reassured, and offered pre-exposure vaccination. Objects, such as utensils, contaminated with patients’ saliva or other secretions should be thoroughly washed with soap, or detergent.

### 4.3. Thirst/Dehydration

Hydrophobic patients cannot tolerate drinking, while those with paralytic rabies often cannot swallow. As a result, these patients may become dehydrated and desperately thirsty. Some may be able to eat fruit such as bananas and suck citrus fruits to combat thirst, and their lips and tongue may be moistened with a damp sponge or flannel. Treatment demands a secure intravenous (iv) line, ideally a catheter rather than a needle. The iv site should be immobilised by splinting. Isotonic 5% glucose, 0.9% saline, or Hartmann’s (Ringer’s lactate) solution (of sodium chloride, sodium lactate, potassium chloride, and calcium chloride) can be used as appropriate. Other possible routes for parenteral rehydration, depending upon available skills and equipment, include intraperitoneal, intraosseous, subcutaneous (sc), or intrarectal (proctoclysis).

### 4.4. Fever

Since physical methods such as tepid sponging and fanning are intolerable to most patients with furious rabies, antipyretic drugs are necessary to control fever. Aspirin, ibuprofen or diclofenac, and paracetamol (acetaminophen) can be given by non-oral routes, such as iv, im, or intrarectal (Table 2). Many patients have evidence of a generalised inflammatory response (e.g. peripheral neutrophil leucocytosis) as a cause for fever [7,8]. However, when the fever is central (neurogenic) in origin, antipyretics may be ineffective. Drugs that have proved effective in individual cases of central hyperthermia include baclofen, bromocriptine, amantadine, dantrolene, and propranolol, but these are unlikely to be widely available in developing countries [30].

### 4.5. Anxiety, Fear, Restlessness, Agitation, Seizures—Use of Sedatives and Tranquillisers

Benzodiazepines are drugs of choice as they are usually available and are on the World Health Organization’s list of essential medicine [31]. They are used widely in daily clinical practice in most places and can be administered by various routes (Table 2). Diazepam can be given in the same doses intramuscularly (im) (which may be painful), intravenously (iv), or intrarectally. Although this is a fatal condition, it is important to avoid depressing respiration by giving the diazepam too rapidly by iv injection. Diazepam will alleviate the patient’s suffering, while giving the family time to adjust and consider the possibility of taking them home to die, if that is their personal or cultural preference [32]. This can be achieved by giving a slow injection (0.5 ml/min, which is 2.5 mg/min) of diazepam. The same doses of diazepam given intrarectally may be useful if the family wish to take the patient home.

Midazolam, an alternative benzodiazepine, has a much shorter half-life. When given im, sc, or iv, it should be ‘titrated’ against the patient’s clinical condition, which must be assessed frequently. Small doses are injected im at frequent intervals or by continuous iv infusion using an electric syringe.

A study of 45 patients in Manila identified their principal distressing symptoms and signs as hydrophobia, aggression, hyperexcitability, aerophobia, disorientation, hypervigilance, difficulty swallowing, hallucination, hypersalivation, anxiety/fear, seizure activity, and restlessness/agitation [33]. Anxiety/fear and restlessness/agitation were relieved by giving either haloperidol alone or diazepam alone, while a combination of the two was also effective against aggression/disorientation/hallucinations. Hypersalivation, hydrophobia and aerophobia were not affected by these treatments. Adult dosages were: haloperidol 5 mg sc or im every hour for at least three doses or until the patient became calm, then haloperidol 5 mg sc or im every 4 or 6 hourly and as needed; and diazepam 20 mg im every 2 h. Haloperidol can also be given by sc injection or infusion and diazepam intrarectally and by iv injection, but only under strict supervision. A patient with rabies encephalomyelitis in Wisconsin, USA, developed neuroleptic malignant syndrome that was attributed to haloperidol [34], but this is too rare a complication of treatment with drugs like haloperidol and chlorpromazine to contraindicate their use in likely-fatal disease. If none of these drugs is available, other possibilities include lorazepam (im, slow iv) or barbiturates (slow iv).

### 4.6. Hypersecretion (Salivation, Lacrimation, Sweating)

This may be reduced by anti-muscarinic anticholinergic drugs, such as hyoscine (scopolamine) hydrobromide, that block parasympathetic secretory activity.

### 4.7. Pain

There is a role for opioids and other powerful analgesics, when they are available, to relieve pain and suffering in rabies victims. Morphine can be given iv, sc, im, or intrarectally, and fentanyl transdermally by patch, which may be especially valuable for terminal management of patients after they have returned home.

As the infection progresses, coma and respiratory, cardiovascular, neurological, endocrine, or gastrointestinal complications will eventually ensue. When the diagnosis is clear, palliative care is the only compassionate strategy for treating previously unvaccinated patients infected by dogs or other terrestrial mammals.

At this stage, neuronal infection will be widespread. Animal experiments show that, even if cells are cleared of the virus, abnormal gene expression remains. Alterations in some neuronal functions would be permanent [35].

## 5. The Likelihood of Recovery Will Vary According to the Origin of Infecting Virus and Whether the Patient Has Been Vaccinated

In the Americas, all bat rabies viruses are rabies genotype/species 1, but are genetically distinct from the classic rabies viruses of terrestrial mammals in the same group [36]. Recovery from encephalomyelitis has been seen in two patients infected by these American insectivorous bat strains that may be less pathogenic in man. Rabies-related lyssaviruses, which infect bats in the rest of the world, show clinical signs similar to classical rabies viruses. Rabies viruses from dogs and other terrestrial mammals have been rapidly fatal in unvaccinated patients.

In patients who have had some rabies vaccine before the onset of symptoms, the progression of the disease can be more gradual. The surprising case of a man bitten by a dog in Turkey and incompletely vaccinated, and who developed rabies, is reported to have shown signs of spontaneous recovery with decreasing hydrophobia, beginning on his second day in hospital. Rabies antibody was present when first tested within a week of the onset of symptoms. He had had supportive treatment but no specialised intensive care and returned to normal life as a shepherd [3].

This case suggests that keeping the patient in hospital with simple clinical management does not prevent recovery if the patient’s immunity is able to control the infection. In contrast, ITU treatment of patients who were vaccinated before the onset of symptoms can prolong the illness, but without any signs of spontaneous improvement, and leave them with severe persistent neurological deficits.

## 6. A Dilemma Arises when Expert ITU Facilities are Available

The decision whether or not to give intensive care relies on confirming the diagnosis in patients at an early stage before they have developed signs of overt clinical rabies, and rapidly testing the neutralising antibody level. If there is a history of an insectivorous bat bite in the Americas, ITU therapy may be considered. However, the diagnosis of rabies might be made only at a terminal stage or post-mortem, because the patients were unaware of any exposure, especially contact with a bat.

Analysis of clinical data leads to the conclusion [37,38] that, if rabies is diagnosed in a previously-vaccinated patient infected by a dog, the relatives must be made aware of the fact that, although intensive care can prolong life, the patient is most unlikely ever to regain full consciousness and will be left with multiple disabilities [39]. It is therefore wise to decide against intensive care for unvaccinated patients infected by a terrestrial mammal in routine clinical practice. This also applies to previously-vaccinated patients with clear signs of rabies who fail to show a rapid steep increase in neutralising antibody levels and early signs of improvement. Progression of the disease despite intensive care indicates that palliation rather than intervention is the better course of action.

Recovery from rabies is extremely rare and associated with the presence of neutralising antibody in the blood and also in the CSF.

ITU therapy could therefore be considered if patients:

1. have been vaccinated previously;
2. develop rabies antibody within the first week of illness;
3. were infected by an American bat rabies virus (see above), especially if they present early.

Alleviating symptoms and signs of respiratory, cardiac, and other complications demands expert ITU management. The aim is to maintain homeostasis until the endogenous antibody and probably cellular immunity generated by the patient inhibits viral replication and clears virus from neurological tissue by unknown mechanisms [1,2].

If the diagnosis were initially in doubt and intensive care has already been started, the pressure to continue may be difficult to resist. However, clinicians should be confident in recognising that the disease is beyond recovery, stop aggressive interventions, and begin palliative care to avoid the patient’s and their relatives’ suffering further.

Striving to keep patients alive without a reasonable prospect of restoring them to a good quality of life would be considered unethical.

## 7. Antiviral Therapy?

At present, there is no specific antiviral agent or other therapy to kill or neutralise rabies virus in the brain. The ‘Milwaukee’ protocol gave rise to much hope but has proved no more effective than expert ITU therapy alone [38]. Several antiviral or immunotherapeutic methods have been suggested or tried [40,41]. One of these, favipiravir, a purine analogue, was effective against other ssRNA viruses. It showed some suppression of street rabies virus replication *in vitro*, but this effect may vary with different neuroblastoma cell lines. When used in mice as oral post-exposure prophylaxis an hour after inoculation as an alternative to rabies immunoglobulin, it reduced mortality and the titre of virus in the brain when the highest dose of 300 mg/kg/day was used. If favipiravir was delayed, starting four days post-inoculation or at the onset of symptoms, there was no effect on mouse mortality [42]. A similar study of favipiravir given to mice soon after inoculation showed no effect on mortality [43].

In the future, however, more effective treatment of rabies encephalitis may become possible. A promising method is injection of an attenuated rabies virus intrathecally, or perhaps iv injection of neutralising antibody, if the blood brain barrier can be bypassed by intraventricular administration [41], or breached pharmacologically [44,45]. Such apparently drastic therapy would be ethical for some sufferers of this fatal infection, but should be undertaken only when there is evidence of effectiveness in animals, and in an ethical and specialised research setting.

Hospitalization often provides little palliative care in developing countries. To correct this deficiency, clear practical guidelines based on recent relevant literature should be made available on national and international websites. The WHO and the World Medical Association are in a good position to promote this compassionate policy.

## 8. Prophylaxis

Rabies vaccine prophylaxis has been 100% successful if pre-exposure immunisation is followed by post-exposure booster vaccination.

Although there are no confirmed cases of rabies transmitted to caregivers, hospital staff and the patients’ relatives will be reassured by the protection afforded by vaccination. Giving rabies vaccine intradermally (id) at multiple sites induces rapid seroconversion, which has been known for decades [46]. To complete a pre-exposure course, a second dose im or id should be given at least seven days later [47].

Giving pre-exposure vaccination to children living in dog rabies-endemic areas would be ideal, but is unaffordable. Pre-exposure vaccination is recommended for anyone, especially children living in dog rabies-endemic areas, if they have access to and can afford it. Vials of vaccine can be shared economically between groups, e.g. families, if injected id [47]. A new syringe and needle must be used for each person. For the vaccinee, this is an insurance against having to pay for full post-exposure vaccination and RIG if exposed for the rest of their lives. If exposure does occur in subsequent years, only wound washing and im or id booster post-exposure vaccination, requiring one or two clinic visits, are needed [47]. Intradermal inoculation of rabies vaccine is not accepted by some authorities, in which case the national protocols should be followed.

## 9. Conclusion

Rabies encephalomyelitis inflicts on its sufferers one of the most agonising deaths imaginable [48]. Unfortunately, the inescapably fatal outcome has discouraged medical staff from active management of patients with rabies encephalomyelitis, distracting them from the imperative of humane palliative care. Consideration of the terminal care of rabies patients has been long delayed and deserves formal attention to minimize the suffering of those dying of this terrible disease. Clinicians must have the courage to offer compassionate palliation whenever the diagnosis is evident.

## Figures and Tables

**Table 1:** Samples and methods recommended for rabies diagnosis in patients

Sample	Purpose	Method
Full thickness skin punch biopsy, including hair follicles^‡^	Antigen detection	IFA test on frozen vertical section ^†^ RT- PCR
Saliva^★^ or throat swab Tears CSF	Virus isolation and Antigen detection	Tissue culture Mouse inoculation test RT- PCR
Serum	Neutralising antibody test	Presence of antibody is diagnostic in unvaccinated patients Take sample on admission to save for comparison 7 days later
CSF	Neutralising antibody test	Test in parallel with serum
Brain post-mortem: Needle necropsy **^¶^** or Autopsy sample brain stem & cerebellum	Virus isolation and Antigen detection	Tissue culture Mouse inoculation test IFA test on impression smear ° RT- PCR

**‡** Highest positivity rate; **^†^** rabies antigen seen in nerve twiglets around the base of hair follicles by immunofluorescence (IFA) test; **^★^** easiest sample for antigen detection, repeat daily at least x 3, until a diagnosis is confirmed; **^¶^** necropsies are taken with a long biopsy needle via the medial canthus of the eye through the superior orbital fissure; via the nose through the ethmoid bone; through the foramen magnum or open fontanelles in children; **°** alternative: a direct rapid immunohistochemical test with biotinylated monoclonal antibodies and light microscopy.

**Table 2:** Drugs for the palliative management of patients with confirmed or strongly suspected rabies encephalomyelitis that are included in the WHO Model List of Essential Medicines 20th List (March 2017) and WHO Model List of Essential Medicines for Children 6th List (March 2017). http://www.who.int/medicines/publications/essentialmedicines/en/ Recommended doses are taken from https://www.bnf.org/products/bnf-online/ and https://bnfc.nice.org.uk/.

Indication	Drug	Route of administration	Dose: adult	Dose: paediatric
**Fever**	paracetamol	iv infusion over 15 minutes	1 g every 4–6 h, maximum 4 g/24 h	
		intrarectal	1 g every 4–6 h, maximum 4 g/24 h	125–500 mg every 4–6 h
	ibuprofen	intrarectal	300–400 mg 6–8 hrly	
	aspirin	intrarectal	450–900 mg 4 hrly, maximum 3.6 g/day	
**Anxiety, agitation, seizures**	diazepam	iv (slow! caution!)	10 mg in 3–5 minutes, repeated 1–4 hrly	0.1–0.3 mg/kg in 3–5 min, repeated 1–4 hrly to provide 2.4–12 mg/kg/24 h
		im (painful!)	20 mg 2 hrly	0.1–0.3 mg/kg 1–4 hrly
		intrarectal	10mg 1–4 hrly	0.1–0.3 mg/kg 1–4 hrly
	lorazepam	im or slow iv injection into large vein (slow! caution!)	25–50 microg/kg 6 hrly	25–50 microg/kg 6 hrly
	midazolam	im	0.08 – 0.2 mg/kg repeated 1–4 hrly	0.07–0.1 mg/kg repeated 1–4 hrly
		iv or sc injection	2.5 mg hrly	
		sc infusion	10–30 mg over 24 h by pump	
		intrarectal		300–500 microg 1–4 hrly
**Anxiety, agitation**	haloperidol	im or sc injection	5 mg hourly until calm, then 4 or 6 hrly and when necessary	age 1 month–12 y: 25–85 microg/kg/24 h
		iv or sc infusion	5–15 mg/24 h	12–18 y: 1.5–5 mg/24 h
	chlorpromazine	deep im	25–50 mg/6–8 hrly	500 microg/kg 6–8 hrly
		intrarectal	100 mg/6–8 hrly	
**Hypersecretion**	hyoscine (scopolamine) hydrobromide	sc or iv injection	400 microg 4 hrly	10 microg/kg 4–8 hrly
		sc infusion	1.2–2 mg/24 h	40–60 microg/kg/24 h
**Pain**	morphine	slow iv, sc or im	10 mg 4 hrly	
		intrarectal	15–30 mg 4 hrly	100 microg/kg
	fentanyl	transdermal patch	12–25 microg/h every 72 h	12 microg/h every 72 h
